# Anticancer Activity of Certain Herbs and Spices on the Cervical Epithelial Carcinoma (HeLa) Cell Line

**DOI:** 10.1155/2012/564927

**Published:** 2012-05-09

**Authors:** Danielle Berrington, Namrita Lall

**Affiliations:** Department of Plant Sciences, University of Pretoria, Pretoria 0002, South Africa

## Abstract

Acetone extracts of selected plant species were evaluated for their *in vitro* cytotoxicity against a noncancerous African green monkey kidney (Vero) cell line and an adenocarcinoma cervical cancer (HeLa) cell line. The plants studied were *Origanum vulgare* L. (Oregano), *Rosmarinus officinalis* L. (Upright and ground cove rosemary), *Lavandula spica* L. (Lavender), *Laurus nobilis* L. (Bay leaf), *Thymus vulgaris* L. (Thyme), *Lavandula x intermedia* L. (Margaret Roberts Lavender), *Petroselinum crispum* Mill. (Curly leaved parsley), *Foeniculum vulgare* Mill. (Fennel), and *Capsicum annuum* L. (Paprika). Antioxidant activity was determined using a quantitative DPPH (1,1-diphenyl-2-picryl hydrazyl) assay. The rosemary species exhibited effective radical scavenging capacity with 50% inhibitory concentration (IC_50_) of 3.48 ± 0.218 *μ*g/mL and 10.84 ± 0.125 *μ*g/mL and vitamin C equivalents of 0.351 g and 1.09 g for McConnell's Blue and Tuscan Blue, respectively. Cytotoxicity was measured using XTT (Sodium 3′-[1-(phenyl amino-carbonyl)-3,4-tetrazolium]-bis-[4-methoxy-6-nitro] benzene sulfonic acid hydrate) colorimetric assay. Only *L. nobilis* and *O. vulgare* exhibited pronounced effects on the HeLa cell line. Dose-dependent studies revealed IC_50_ of 34.46 ± 0.48 *μ*g/mL and 126.3 ± 1.00 *μ*g/mL on the HeLa cells and on the Vero cells 124.1 *μ*g/mL ± 18.26 and 163.8 *μ*g/mL ± 2.95 for *L. nobilis* and *O. vulgare*, respectively. Light (eosin and haematoxylin staining) and confocal microscopy (Hoechst 33342, acridine orange, and propidium iodide staining) were used to evaluate the cytotoxic mechanism of action for *L. nobilis* and *O. vulgare*.

## 1. Introduction

South Africa has a remarkable floral and cultural diversity with an estimate of 30,000 species of higher plants, which are mostly endemic to the region. A great number of these species are used for traditional medicinal purposes where Zulu traditional healers use around 1032 different species from 147 various plant families [1]. It is estimated that around 27 million South African citizens rely on these traditional medicines for their primary health care needs due to the inaccessibility of western medicine to some regions and the high cost [2]. Therefore, the demand for traditional medicines remains high in rural parts [1].

According to the World Health Organization (WHO) cancer is one of the leading causes of death worldwide, which accounted for 7.6 million deaths (around 13%) of the world's population in 2008. They have furthermore estimated that the worldwide deaths are likely to rise to over 11 million in 2030. The Cancer Association of South Africa (CANSA) has estimated that one in every six South African males and one in every seven South African females are diagnosed with cancer. One of the most prevalent types of cancer found in South African females is cervical cancer which is also the most widespread cancer found in South African black women and in poor-income countries.

Cervical cancer is caused by the Human Papilloma Virus (HPV) which forms warts in the throat and genital area. Although it is the most prevalent cancer in South African black women it is also the most preventable cancer through screening and treatment of precancerous lesion and the use of vaccines. Natural products are being tested for the treatment of cancer as conventional cancer treatments such as chemotherapy destroy cancerous as well as healthy cells.

In South Africa the use of plants for the treatment of cancer is not common [3]. There is, however, a Southern African plant currently undergoing clinical trials for the treatment of cancer. The plant is *Combretum caffrum*, more commonly known as the “bush willow” which causes the shutdown of tumours and tumour necrosis [4]. Herbs and spices have been used for thousands of years as medicines. Through scientific research it is indicated that the bioactive components present in herbs and spices can reduce the risk of cancer through their antimicrobial, antioxidant, and antitumorogenic activity and their ability to directly suppress carcinogen bioactivation [5]. In this study common herbs and spices were tested for their cytotoxic activity on cervical cancer (HeLa) cells. These plants were chosen as they are readily available in South Africa and because they are traditionally used all over Africa. This study will be used as a comparative study on future research which will focus mainly on indigenous South African plants and their cytotoxic activity on cervical cancer cells.

## 2. Materials and Methods

### 2.1. Chemicals and Reagents

 DPPH, ascorbic acid, Bouin's fixative, haematoxylin, eosin, xylene, Hoechst 33342, propidium iodide, and N-propyl-gallate were of analytical grade and supplied by Sigma Aldrich (St. Louis, MO, USA). All cell lines, media, trypsin-EDTA, fetal bovine serum (FBS), phosphate buffer saline (PBS), antibiotics, and glycerol were supplied by Highveld Biological (Pty) Ltd. (Modderfontein, Johannesburg, RSA).

### 2.2. Plant Material

 All plant material was collected during February 2011 in Pretoria, Gauteng. The plants were identified by the University of Pretoria Botanical Garden curator, Jason Sampson. The collected aerial parts of the plants were shade-dried for two weeks.

### 2.3. Preparation of Acetone Extracts

 The air-dried aerial parts of the plant were mechanically ground to produce a fine powder. The weighed samples were extracted with acetone for 48 h and thereafter for 24 h using fresh solvent. The solute of each plant was filtered with a Buchner funnel. Thereafter the menstruum was evaporated to dryness using a vacuum rotary evaporator to give a dark green extract.

### 2.4. Antioxidant Activity—DPPH Assay

 The method of du Toit et al. [6] was followed to determine the radical scavenging capacity (RSC) of the extracts. Slight modifications to the method were made and are described briefly. Stock solutions of vitamin C and the extracts were prepared at concentrations of 2 mg/mL and 10 mg/mL, respectively. To each well in the top row of a 96-well plate, 200 *μ*L of distilled water was added. To the rest of the wells 110 *μ*L of distilled water was added as a medium. Twenty microlitres of extract was added to the first top wells, in triplicate, followed by serial dilution with final concentrations ranging from 3.9 *μ*g/mL to 500 *μ*g/mL for the extracts and 0.781 *μ*g/mL to 100 *μ*g/mL for vitamin C [7]. Finally 90 *μ*L of 0.04 M DPPH ethanolic solution was added to each well, except for the negative control where distilled water was added instead. The plates were left in a dark room to develop for 30 minutes. The RSC of the extracts was determined using a BIO-TEK Power-Wave XS multiplate reader at a wavelength of 515 nm, using KC junior software. The IC_50_ values of each extract were calculated using GraphPad Prism 4 software. Lastly, the vitamin C equivalent for each extract was calculated as follows: (IC_50_ of extract × 200 mg vitamin C)/IC_50_ of vitamin C.

### 2.5. Cell Culture

 The HeLa and Vero cell lines were maintained in culture flasks containing Eagle's Minimum Essentail Medium supplemented with 10% heat-inactivated FBS and 1% antibiotics (100 U/mL penicillin, 100 *μ*g/mL streptomycin and 250 *μ*g/mL fungizone). The cells were grown at 37°C in a humidified incubator set at 5% CO_2_. Cells were subcultured after they formed a monolayer on the flask. The cells were detached by treating them with trypsin-EDTA (0.25% trypsin containing 0.01% EDTA) for 10 minutes and then by adding complete medium to inhibit the reaction.

### 2.6. *In Vitro* Cytotoxicity Assay

 Cytotoxicity was measured by the XTT method using the Cell Proliferation Kit II. The method described by Zheng et al. [8] was used to perform the assay. Both HeLa and Vero cells were seeded (100 *μ*L) in a 96-well microtitre plate (concentration 1 × 10^5^ cells/mL). The plate was then incubated for 24 h at 37°C and 5% CO_2_ to allow the cells to attach to the bottom of the wells. The extracts were each prepared to a stock solution of 20 mg/mL and added to the microtitre plate. Serial dilutions were made to range from a concentration of 400 *μ*g/mL–1.563 *μ*g/mL for each extract. The microtitre plate was incubated for a further 72 h. The control wells included vehicle-treated cells exposed to 2% DMSO (sample concentration of 400 *μ*g/mL) and the positive control Actinomycin D with concentrations ranging between 0.5 *μ*g/mL and 0.002 *μ*g/mL. After the 72 h incubation period, the XTT reagent (50 *μ*L) was added to a final concentration of 0.3 mg/mL and the plate was then further incubated for another 2 hours. After the incubation the absorbance of the colour complex was read at 490 nm with a reference wavelength set at 690 nm using a BIO-TEK Power-Wave XS multiwell plate reader. The assay was performed in triplicate to calculate an IC_50_ of the cell population for each extract. The results were analysed using the GraphPad Prism 4 program.

### 2.7. Light Microscopy (Haematoxylin and Eosin Staining)

 The cancerous HeLa cells were exposed to 34.46 *μ*g/mL (IC_50_) and 68.92 *μ*g/mL (2IC_50_) of the *Laurus nobilis* extract and 126.3 *μ*g/mL (IC_50_) and 252.6 *μ*g/mL (2IC_50_) of the *Origanum vulgare* extract. The Vero cells were exposed to concentrations of 124.1 *μ*g/mL (IC_50_), 248.2 *μ*g/mL (2IC_50_) and 34.46 *μ*g/mL (HeLa IC_50_) for *Laurus nobilis* and concentrations of 163.8 *μ*g/mL (IC_50_), 327.6 *μ*g/mL (2IC_50_), and 126.3 *μ*g/mL (HeLa IC_50_) for *Origanum vulgare*. The concentrations were chosen due to their antiproliferative activity that was observed at these concentrations. Cells were seeded at 100,000 cells per well in a 6-well plate on heat-sterilized cover slips and incubated for 24 h at 37°C at 5% CO_2_ for cell adherence. The cells were then exposed to the above concentrations including a vehicle-treated control (2% DMSO), actinomycin D (0.05 *μ*g/mL) as well as cells propagated in growth medium. The cells were incubated for a further 72 h at 37°C. After 72 h the cells were fixed with Bouin's fixative for 30 min and stained by standard haematoxylin and eosin procedures [9]. Briefly, the cells were rinsed in 70% ethanol for 20 min and thereafter stained in haematoxylin for a further 20 min. The cells were rinsed again and stained for 2 min in 1% eosin solution. After several rinsing procedures with different percentages of ethanol, the cells were left in xylene for 10 min. The cover slips were mounted to the microscope slides with the remaining resin and evaluated using a Nikon Stereo Light microscope equipped with a 1.4 Apo oil lens under 400x magnification.

### 2.8. Confocal Microscopy (Hoechst 33342 and Propidium Iodide Staining)

 A double fluorescent dye staining method was used according to Stander et al. [9]. Exponentially growing cells were seeded at 100,000 cells per well in a 6-well plate onto heat-sterilized cover slips and incubated for 24 h at 37°C and 5% CO_2_ to allow the cells to attach. After the incubation period the medium was discarded and the cells were exposed to the same concentrations as used in Section 2.7 and incubated for a further 72 h. After 72 h the treatment was inhibited by discarding the extract and washing the cells with PBS. The cells were stained with 0.5 mL of Hoechst 33342 solution (3.5 *μ*g/mL in PBS) and incubated for 30 min at 37°C incubator. After 30 min the Hoechst 33342 solution was discarded and the cells were then stained with 0.5 mL of propidium iodide (40 *μ*g/mL in PBS) and incubated for a further 5 min at 37°C. Thereafter the cover slips were mounted on microscope slides with mounting fluid (90% glycerol, 4% N-propyl-gallate, 6% PBS) and observed under a confocal microscope (ZEISS, LSM 510 Meta confocal) using 1000x magnification.

Microscopy studies were done in triplicate so that the appearance of apoptosis or normal cell proliferation could be confirmed. An alternative is that blind sampling can be done whereby microscope slides are numbered and viewed under the microscope without knowing which treatment was applied and thereafter confirming which treatment was done.

## 3. Results and Discussion

### 3.1. Determination of Antioxidant Activity

**Figure 1:**
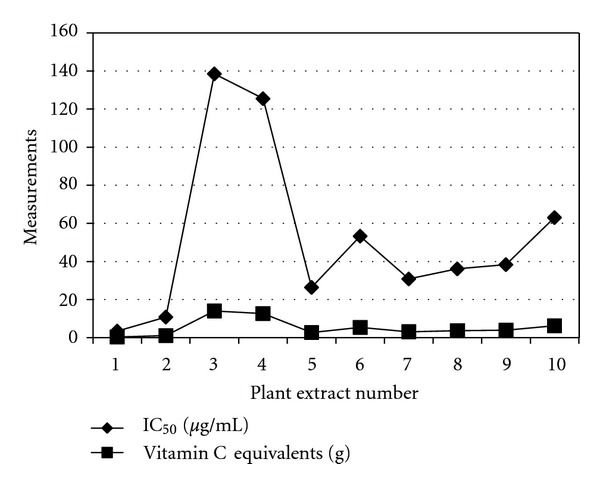
Comparison of the IC_50_ values and the Vitamin C equivalents of the plant extracts.

 All extracts showed dose-dependent responses with concentrations ranging from 500 *μ*g/mL to 3.90 *μ*g/mL. The highest inhibition was shown at 500 *μ*g/mL and the least inhibition at 3.90 *μ*g/mL. The rosemary species showed the most effective RSC as recorded in Table 1. Bubonja-Sonje et al. [10] conducted a study on the extracts of *Rosmarinus officinalis* L. to test for their free radical scavenging activity of DPPH. These extracts of rosemary were prepared from a methanol : water : acetic acid (90 : 9 : 1, v/v/v) solvent. The antioxidant content was attributed to the fact that rosemary contained a very rich source of polyphenolic compounds, more specifically phenolic diterpenes, such as Carnosol and Carnosic acid. Specifically carnosic acid was tested for its antioxidant activity and was determined to have an IC_50_ value of 27.3 ± 5.2 *μ*g/mL. In the present study the antioxidant activity of the McConnell's Blue and Tuscan Blue extracts were found to be better than that of the rosemary extract investigated by Bubonja-Sonje et al. [10]. The lavender species showed minimal inhibition of DPPH for both for *Lavandula x intermedia* and *Lavandula spica*. In a study conducted by Gülçin et al. [11] aerial parts of the plant *Lavandula stoechas* L. were used to determine the free radical scavenging activity. This part of the plant contained oleanolic acid, ursolic acid, vergatic acid, *β*-sitosterol, *α*-amyrin, *α*-amyrin acetate, lupeol, erythrodiol, flavanoids, luteolin, acacetin, and vitexin. They found that the ethanol extract had an IC_50_ of 60 *μ*g/mL, which was notably much more effective than the IC_50_ values of *Lavandula-x-intermedia* and *Lavandula spica.* As no previous studies have been reported on the antioxidant content of *Lavandula-x-intermedia* and *Lavandula spica* it is difficult to determine whether the compounds present in *Lavandula stoechas* L. are also present in the extracts used in the present study. The other plant materials had RSC ranging from good to relatively good which can be seen in Table 1 and Figure 1 together with the vitamin C equivalents for each extract. The vitamin C equivalent is a measurement of the amount extract (g) that would be needed to have a RSC that is equivalent to the RSC of 200 mg vitamin C. The vitamin C equivalents were calculated according to the following equation: (IC_50_ extract × 200 mg vitamin C)/IC_50_ vitamin C. The positive control vitamin C had an IC_50_ of 1.98 ± 0.006 *μ*g/mL.

### 3.2. Cytotoxicity of Extracts

 All plant materials were tested for their anticancer activity using the XTT colorimetric assay. The plant extracts were prepared and screened for their *in vitro* cytotoxic effects against HeLa and Vero cells. The assay is based on the ability of living cells to reduce the yellow-water soluble XTT into an insoluble formazan product [12]. The IC_50_ values were used to determine the selectivity indexes (SI) of each extract which represents the overall activity. SI values were calculated as follows: (IC_50_ of Vero/IC_50_ of HeLa). The cytotoxicity and SI of the ten plant extracts are shown in Table 2.

*Laurus nobilis* and *Rosmarinus officinalis* (Tuscan Blue) strongly inhibited the proliferation of the HeLa cells as seen in Table 2. The lowest toxicity was observed from the *Thymus vulgaris* and *Capsicum annuum* (Paprika) extracts. All other extracts had IC_50_ values ranging from 98.99 ± 1.28 *μ*g/mL to 182 ± 0.15 *μ*g/mL. The IC_50_ value of *Origanum vulgare* was noted and was used in further mechanistic studies together with *Laurus nobilis*. Actinomycin D, a known anticancer agent, had an IC_50_ value of 0.002 ± 0.0000395 *μ*g/mL. The toxicity on the Vero cells ranged from a high toxicity with an IC_50_ value of 12.03 ± 0.02 *μ*g/mL for *Rosmarinus officinalis* (McConnell's Blue) to a low toxicity with an IC_50_ value of 163.8 ± 2.92 *μ*g/mL for *Origanum vulgare*. It is interesting to note that the two rosemary species exhibited varying cytotoxicity against the HeLa cells. The anticarcinogenic activity of rosemary is due to the major bioactive compounds such as rosmarinic acid, carnosic acid, and carnosol [13]. Tuscan blue rosemary could possibly have a higher concentration of these bioactive compounds and therefore shows higher toxicity on the HeLa cells. In a similar study where compounds extracted from *Rosmarinus officinalis* were tested on various cancer cell lines, such as NCI-H82 (small lung carcinoma), DU-145 (prostate carcinoma), Hep3D (liver carcinoma), K-562 (chronic myelois carcinoma), MCF-7, (breast adenocarcinoma), PC-3 (prostate adenocarcinoma) and MDA-MB-231 (breast adenocarcinoma) the IC_50_ values ranged from 8.82 *μ*g/mL to over 100 *μ*g/mL [14]. The lowest toxicity towards the HeLa cell line was seen in the *Capsicum annuum* and *Thymus vulgaris* extracts. In an article published by Cancer Research in *Capsicum* was reported to help in the treatment of prostate cancer. Furthermore the essential oil of *Thymus vulgaris* was tested on head and neck squamous cell carcinoma (HNSCC) by Sertel et al. [15] and was found to have a very low toxicity with an IC_50_ value of 369 *μ*g/mL. The positive control actinomycin D showed an IC_50_ value of 0.027 ± 0.00021 *μ*g/mL.

Extracts which showed SI values equal or greater than one were chosen for further mechanistic studies. From the SI values summarized in Table 2 it was noted that *Origanum vulgare* and *Laurus nobilis* had SI values which were greater than one. When considering the affectivity of these extracts on HeLa cells it was noted that *Laurus nobilis* had the highest toxicity, whereas *Origanum vulgare* had a much lower toxicity. When considering the toxicity of these extracts towards the Vero cells, it is preferred that there is very low toxicity as these cells are used as a comparison to normal human cells. *Origanum vulgare* had the lowest toxicity, whereas *Laurus nobilis* had a slightly higher toxicity. Both these extracts were chosen for further mechanistic studies due to their SI values; however *Origanum vulgare* is not very effective against the HeLa cell line. However in a study conducted by Al-Kalaldeh et al. [16] the volatile oil extract of *Origanum vulgare* was very effective against human breast adenocarcinoma cells (MCF7) with an IC_50_ of 30.1 ± 1.14 *μ*g/mL. In the same study an ethanolic extract of *Laurus nobilis* showed an IC_50_ of 24.49 *μ*g/mL.

### 3.3. Light Microscopy

**Figure 2:**
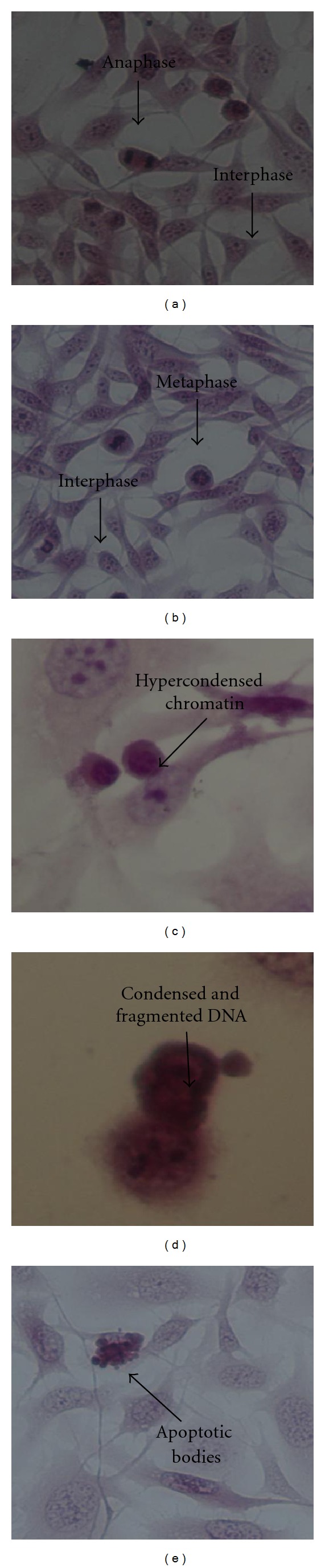
Haematoxylin and eosin staining of HeLa medium only control cells (a), vehicle-treated control cells (b), 34.46 *μ*g/mL *Laurus nobilis* treated cells (c), 68.92 *μ*g/mL *Laurus nobilis* treated cells (d), and 0.05 *μ*g/mL actinomycin D (e), after 72 h of exposure (400x magnification).

*Laurus nobilis* and *Origanum vulgare* were qualitatively analyzed at their IC_50_ and 2IC_50_ values to investigate their influence on the HeLa cells and on the Vero cells. The mechanism of action of both extracts was determined by observing the change in cell morphology after the exposure of the cell lines to the extracts and comparing them to vehicle-treated controls and medium controls. In Figures 2(a) and 2(b) where the cells were propagated in medium and in the presence of DMSO (vehicle-treated), respectively, normal proliferation seemed to take place and typical morphology was observed. In Figure 2(a) anaphase and interphase was observed whereas in Figure 2(b) metaphase and interphase was observed, which are typical characteristics of the cell cycle. However in Figures 2(c) and 2(d) normal cell proliferation was absent and signs of cell death and growth disorders started to appear. In these photographs the HeLa cells were exposed to the IC_50_ and 2IC_50_ values of *Laurus nobilis* extract. In both these photographs typical morphological features of apoptosis started to appear such as hypercondensed chromatin and the degredation of DNA. The positive control actinomycin D also showed features of apoptosis, such as the presence of apoptotic bodies (Figure 2(e)).

**Figure 3:**
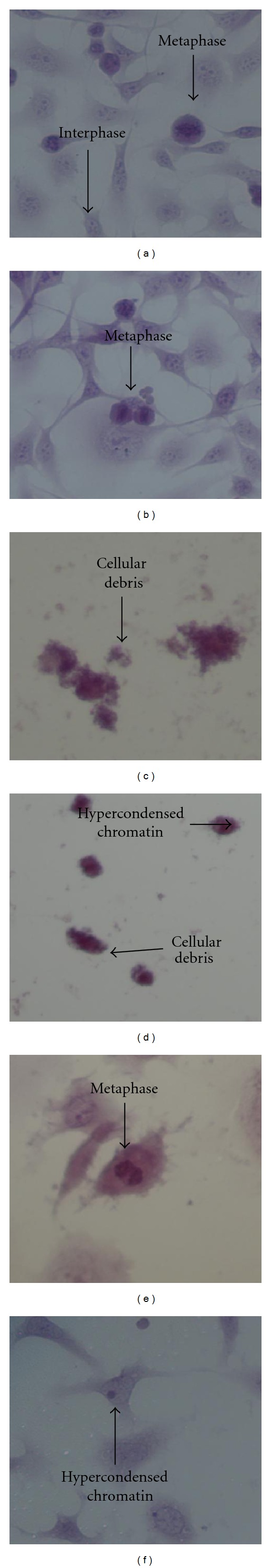
Haematoxylin and eosin staining of Vero medium only control cells (a), vehicle-treated control cells (b), 124.1 *μ*g/mL *Laurus nobilis* treated cells (c), 248.2 *μ*g/mL *Laurus nobilis* treated cells (d), 34.46 *μ*g/mL *Laurus nobilis* treated cells (e), and 0.05 *μ*g/mL actinomycin D (f), after 72 h of exposure (400x magnification).

Observing the Vero cells in the absence and presence of *Laurus nobilis* and actinomycin D (Figure 3) similar morphological changes were seen as in Figure 2. When the cells were grown in medium only and in the presence of DMSO normal cell proliferation took place such as metaphase (Figures 3(a) and 3(b)) and interphase (Figure 3(a)). In the photographs where the cells were exposed to the IC_50_ and 2IC_50_ values of *Laurus nobilis* changes in cellular morphology started to take place. In both Figures 3(c) and 3(d) cellular debris was observed which could be as a result of lysis of apoptotic bodies. Furthermore the presence of hypercondensed chromatin was seen in Figure 3(d). In the presence of actinomycin D (Figure 3(f)) hypercondensed chromatin was also visible. An important observation was made in Figure 3(e) where the Vero cells were treated with the IC_50_ dose of *Laurus nobilis* on HeLa cells. In this photograph normal proliferation took place as seen by the presence of metaphase. It was important to observe that the Vero cells revealed less prominent features of cell death when they were treated at the IC_50_ of *Laurus nobilis* on HeLa cells.

**Figure 4:**
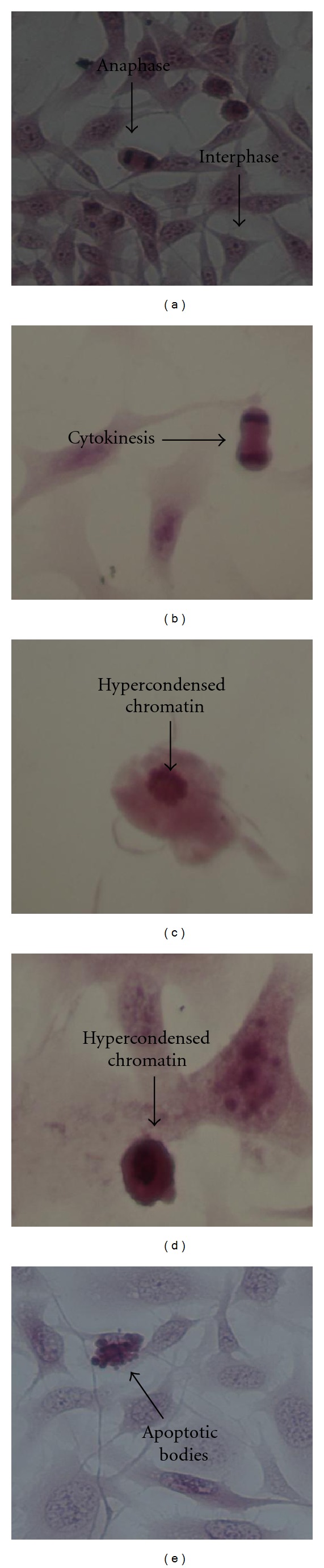
Haematoxylin and eosin staining of HeLa medium only control cells (a), vehicle-treated control cells (b), 126.3 *μ*g/mL *Origanum vulgare* treated cells (c), 252.6 *μ*g/mL *Origanum vulgare* treated cells (d), and 0.05 *μ*g/mL actinomycin D (e), after 72 h of exposure (400x magnification).

In the HeLa cell line when the cells were grown in medium only and exposed to DMSO, normal cell cycle morphology was viewed. In Figure 4(a) anaphase was observed whereas in Figure 4(b) cytokinesis was observed, which is the last step in normal cell cycle before two daughter cells are formed. In Figures 4(c) and 4(d) when cells were treated at the IC_50_ and 2IC_50_ values of *Origanum vulgare*, apoptotic morphological features were viewed such as hypercondensed chromatin and in the presence of actinomycin D apoptotic bodies were observed. Therefore when HeLa cells were treated with the *Origanum vulgare* extract there was an increase in morphological features which correlated with apoptosis.

**Figure 5:**
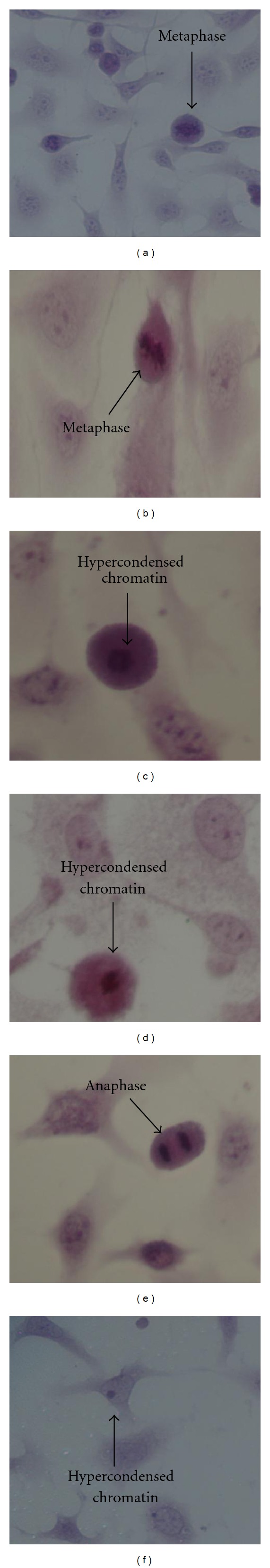
Haematoxylin and eosin staining of Vero medium only control cells (a), vehicle-treated control cells (b), 163.8 *μ*g/mL *Origanum vulgare* treated cells (c), 327.6 *μ*g/mL *Origanum vulgare* treated cells (d), 126.3 *μ*g/mL *Origanum vulgare* treated cells (e), and 0.05 *μ*g/mL actinomycin D (f), after 72 h of exposure (400x magnification).

In the Vero cell line, vehicle-treated controls (Figure 5(b)) and medium-only controls (Figure 5(a)) were viable and still showed signs of proliferation as seen by the presence of metaphase in both photographs. Actinomycin D (Figure 5(f)) as well as cells treated with 163.8 *μ*g/mL and 327.6 *μ*g/mL of the *Origanum vulgare* extract (Figures 5(c) and 5(d)) showed prominent signs of cell death occurring through apoptosis such as hypercondensed chromatin. In Figure 5(e) where the Vero cells were treated with 126.3 *μ*g/mL of *Origanum vulgare* normal cell proliferation took place where anaphase was observed. This concentration is where 50% of the HeLa cells were inhibited by the extract, but showed less prominent and negligible signs of toxicity to the noncancerous cell line.

### 3.4. Confocal Microscopy

**Figure 6:**
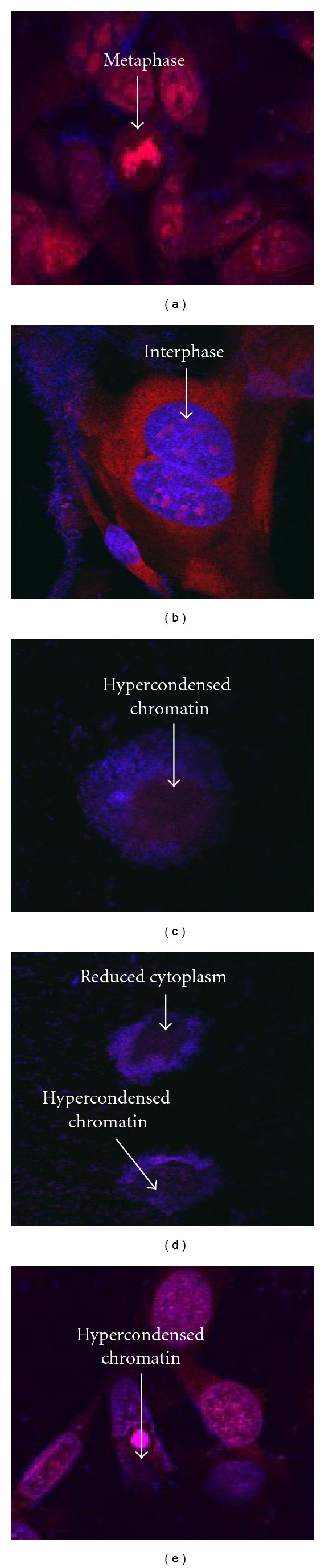
Hoechst 33342 and propidium iodide staining of HeLa medium only control cells (a), vehicle-treated control cells (b), 34.46 *μ*g/mL *Laurus nobilis* treated cells (c), 68.92 *μ*g/mL *Laurus nobilis* treated cells (d), and 0.05 *μ*g/mL actinomycin D (e), after 72 h of exposure (1000x magnification).

 Change in morphological features when the cancerous (HeLa) and noncancerous (Vero) cell lines were exposed to various concentrations of the acetone extracts of *Laurus nobilis* and *Origanum vulgare* were confirmed when visualizing the cells under confocal microscopy. The HeLa cells showed normal signs of cell proliferation when grown in medium only or when exposed to 2% DMSO as seen in Figures 6(a) and 6(b) respectively. Normal cell growth and proliferation was confirmed by the presence of metaphase and anaphase. Actinomycin D (Figure 6(e)) showed clear signs of cell death with the presence of hypercondensed chromatin. *Laurus nobilis* treated HeLa cells revealed an increase in morphological features which were characteristic of apoptosis, which included the presence of hypercondensed chromatin and reduced cytoplasm (Figures 6(c) and 6(d)). The reduction in cytoplasm could have been due to the detachment of cells during the time of incubation.

**Figure 7:**
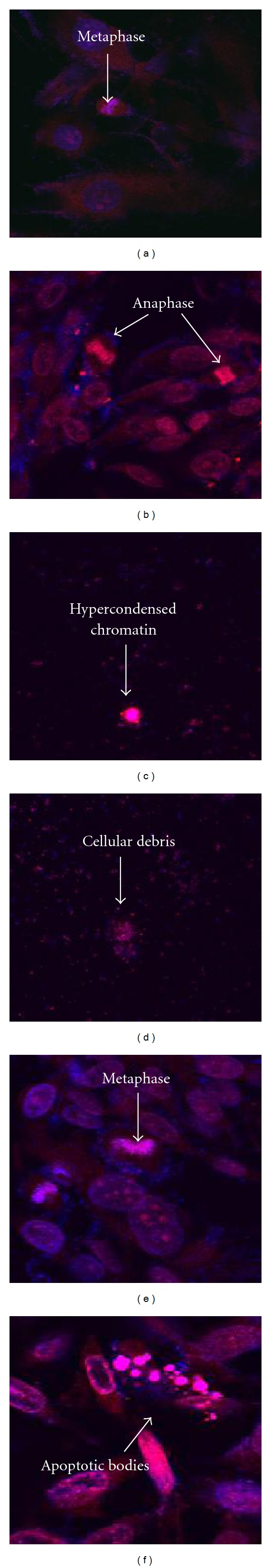
Hoechst 33342 and propidium iodide staining of Vero medium only control cells (a), vehicle-treated control cells (b), 124.1 *μ*g/mL *Laurus nobilis* treated cells (c), 248.2 *μ*g/mL *Laurus nobilis* treated cells (d), 34.46 *μ*g/mL *Laurus nobilis* treated cells (e), and 0.05 *μ*g/mL actinomycin D (f), after 72 h of exposure (1000x magnification).

In the Vero cells normal cell cycle features such as metaphase (Figure 7(a)) and anaphase (Figure 7(b)) were observed indicating that there were no growth deformities. However in Figures 7(c) and 7(d) changes in morphology started to appear such as the presence of hypercondensed chromatin and cellular debris. Furthermore in the presence of actinomycin D apoptotic bodies started to appear (Figure 7(f)). However in Figure 7(e) normal cell cycle features were viewed such as metaphase which indicates that at the IC_50_ concentration of *Laurus nobilis* on HeLa cells there were less prominent cellular features of cell death on Vero cells.

**Figure 8:**
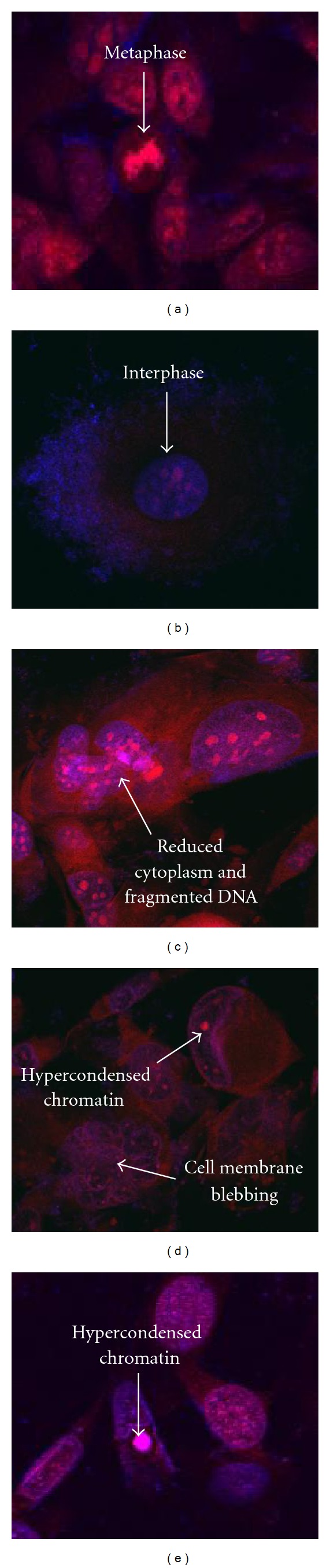
Hoechst 33342 and propidium staining of HeLa medium only control cells (a), vehicle-treated control cells (b), 126.3 *μ*g/mL *Origanum vulgare* treated cells (c), 252.6 *μ*g/mL *Origanum vulgare* treated cells (d), and 0.05 *μ*g/mL actinomycin D (e), after 72 h of exposure (1000x magnification).

In the presence and absence of *Origanum vulgare* on HeLa cells different morphological features were observed. In untreated cells or cells treated with 2% DMSO prominent features corresponding with normal cell growth and proliferation was observed, which included metaphase (Figure 8(a)) and interphase (Figure 8(b)). However in cells treated with 126.3 *μ*g/mL and 252.6 *μ*g/mL of *Origanum vulgare* severe signs of cell death were viewed. In cells treated with an IC_50_ concentration features such as reduced cytoplasm and fragmented DNA was seen (Figure 8(c)), whereas in cells treated at a 2IC_50_ concentration features appeared such as hypercondensed chromatin and cellular membrane blebbing (Figure 8(d)). These features tend to be characteristics of apoptotic cell death. Actinomycin D (Figure 8(e)) showed similar signs of apoptosis with the appearance of hypercondensed chromatin.

**Figure 9:**
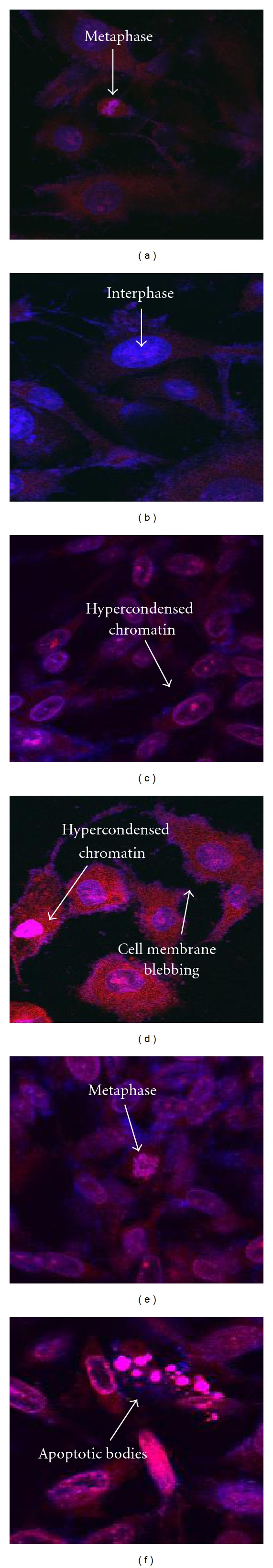
Hoechst 33342 and propidium iodide staining of Vero medium only control cells (a), vehicle-treated control cells (b), 163.8 *μ*g/mL *Origanum vulgare* treated cells (c), 327.6 *μ*g/mL *Origanum vulgare* treated cells (d), 126.3 *μ*g/mL *Origanum vulgare* treated cells (e), and 0.05 *μ*g/mL actinomycin D (f), after 72 h of exposure (1000x magnification).

Vero cells showed normal cell proliferation when exposed to medium only or 2% DMSO treated cells. Cell cycle features such as metaphase (Figure 9(a)) and interphase (Figure 9(b)) were observed. Cells exposed to IC_50_ and 2IC_50_ concentrations showed distinct signs of apoptosis such as hypercondensed chromatin (Figures 9(c) and 9(d)) and cellular membrane blebbing (Figure 9(d)). In Figure 9(e) normal cell proliferation was observed as seen by the presence of metaphase. Actinomycin D also showed morphological features which were characteristic of apoptosis such as apoptotic bodies (Figure 9(f)).

The two microscopy techniques were in agreement with one another. The haematoxylin and eosin staining is based on differentiating the nuclei from the cytoplasm. The haematoxylin stains the nuclei blue/purple whereas the eosin stains the cytoplasm red. The confocal microscopy is based on differentiating intact cells from those that have lost their membrane integrity. The Hoechst 33342 penetrates intact cell membranes of viable cells and those undergoing apoptosis and therefore stains the nucleus. Propidium iodide can only stain the nucleus of cells that have lost their membrane integrity and therefore stain the nucleus of cells undergoing late apoptosis and necrosis [9]. In both cell lines the extracts showed morphological changes including apoptotic bodies, hypercondensed chromatin, reduced cytoplasm, and cellular debris which were indicative of possible apoptosis taking place [17, 18]. In the HeLa cell line the common feature of apoptosis occurring was hypercondensed chromatin whereas in the Vero cell line cellular debris as well as hypercondensed chromatin seemed to occur more often.

## 4. Conclusion

In this study common herbs and spices were chosen to determine their anticancer activity. Herbs and spices were chosen due to their bioactive components which have the ability to reduce the risk of cancer through their antimicrobial, antioxidant, and antitumourogenic activity [19]. The rosemary species were found to have the highest antioxidant content which could be attributed to the high content of polyphenolic compounds. Therefore the rosemary species possess potential chemopreventive properties. After performing the XTT cytotoxicity assay it was hypothesized that *Laurus nobilis*, with an SI of 3.61, and *Oregano vulgare*, with an SI of 1.30, were the best candidates for further investigation. Both these extracts were further investigated to determine their mechanism of cell death by observing them under light microscopy and confocal microscopy. From the microscopy images it was observed that in both cell lines morphological changes did appear after exposure to various concentrations of the acetone extracts. Although these microscopy images were in agreement with one another that apoptosis was the possible mechanism of cell death, as seen by the characteristic features of hypercondensed chromatin (mostly in HeLa cells) and cellular debris (seen only in Vero cells), they are not sufficient enough to definitely confirm that apoptosis is taking place and therefore more sensitive assays such as flow cytometry need to Be used to confirm that apoptosis is taking place. However *Laurus nobilis* did show promising results as an anticancer agent due to its relatively high toxicity on the HeLa cell line and at this same concentration a low toxicity on the Vero cell line.

## Figures and Tables

**Table 1:** A summary of the IC_50_ values of the plant extracts and their vitamin C equivalents.

Plant extracts	IC_50_ values (*μ*g/mL) ± SD	Vitamin C equivalents for a 200 mg capsule (in g)
(1) *Rosmarinus officinalis* (McConnell's Blue)	3.48 ± 0.22	0.351
(2) *Rosmarinus officinalis* (Tuscan Blue)	10.84 ± 0.12	1.09
(3) *Lavandula-x-intermedia* (Margaret Roberts)	138.5 ± 4.0	14.0
(4) *Lavandula spica *	125.5 ± 1.8	12.67
(5) *Origanum vulgare *	26.43 ± 1.58	2.67
(6) *Petroselinum crispum *	53.3 ± 2.63	5.38
(7) *Laurus nobilis *	30.85 ± 0.48	3.12
(8) *Thymus vulgaris *	36.13 ± 0.85	3.65
(9) *Foeniculum vulgaris *	38.36 ± 0.76	3.88
(10) *Capsicum annuum* (Paprika)	63.06 ± 1.91	6.27
(11) Vitamin C^a^	1.98 ± 0.01	—

^a^Positive control for antioxidant assay.

**Table 2:** Summary of the IC_50_ values and selectivity indexes of the plant extracts.

Plant extracts	HeLa IC_50_^a^ (*μ*g/mL) ± SD	Vero IC_50_ (*μ*g/mL) ± SD	SI^b^
*Rosmarinus officinalis* (McConnell's Blue)	>100	12.03 ± 0.02	0.12
*Rosmarinus officinalis* (Tuscan Blue)	23.31 ± 0.055	23.64 ± 0.10	0.52
*Lavandula-x-intermedia* (Margaret Roberts)	182 ± 1.5	31.5 ± 0.14	0.17
*Lavandulaspica*	98.99 ± 1.28	26.3 ± 0.64	0.27
*Origanumvulgare*	126.3 ± 1.00	163.8 ± 2.95	1.30
*Petroselinumcrispum*	172.7 ± 0.25	105 ± 0.50	0.61
*Laurusnobilis*	34.46 ± 0.48	124.1 ± 18.8	3.61
*Thymusvulgaris*	>200	138.4 ± 2.60	0.66
*Foeniculumvulgare*	129.7 ± 2.05	85.37 ± 5.26	0.66
*Capsicum annuum* (Paprika)	>200	122.3 ± 3.76	0.58
Actinomycin D^c^	0.002	0.027	13.5

^a^Fifty percent inhibitory concentration.

^b^Selectivity index.

^c^Positive control for cytotoxicity assay.
